# Supplementary material for: Mechanisms governing the pioneering and redistribution capabilities of the non-classical pioneer PU.1
Source: Nat Commun. 2020 Jan 21;11:402. doi: 10.1038/s41467-019-13960-2 (PMC6972792; doi:10.1038/s41467-019-13960-2)
Supplement: Supplementary file 7 — Source data [file 41467_2019_13960_MOESM7_ESM.zip › Source_Data/FigureS2/FigureS2B_MotifScanOutput/KmeansCluster2/homerResults/motif50.similar.html]

motif50

## Information for motif50

C
T
G
A
C
T
A
G
G
C
T
A
C
T
A
G
G
A
C
T
A
C
G
T
T
C
G
A
T
G
A
C
G
T
A
C
G
T
C
A
A
G
C
T
G
T
C
A
G
A
C
T
  
Reverse Opposite:  

C
T
G
A
C
A
G
T
C
T
G
A
A
C
G
T
A
C
T
G
A
C
T
G
A
G
C
T
G
T
C
A
C
T
G
A
G
A
T
C
C
A
G
T
A
G
T
C
G
A
C
T
  

|  |  |
| --- | --- |
| p-value: | 1e-20 |
| log p-value: | -4.616e+01 |
| Information Content per bp: | 1.739 |
| Number of Target Sequences with motif | 39.0 |
| Percentage of Target Sequences with motif | 0.78% |
| Number of Background Sequences with motif | 28.6 |
| Percentage of Background Sequences with motif | 0.07% |
| Average Position of motif in Targets | 93.0 +/- 56.9bp |
| Average Position of motif in Background | 100.8 +/- 41.8bp |
| Strand Bias (log2 ratio + to - strand density) | -0.2 |
| Multiplicity (# of sites on avg that occur together) | 1.00 |
| Motif File: | file (matrix) reverse opposite |

### Similar de novo motifs found

|  |  |  |  |  |  |  |  |
| --- | --- | --- | --- | --- | --- | --- | --- |
| Rank | Match Score | Redundant Motif | P-value | log P-value | % of Targets | % of Background | Motif file |
| 1 | 0.952 | C T A G C T G A C T A G A G C T A C G T C T G A G A T C A G T C C G T A A G C T T C G A A C G T | 1e-18 | -43.129513 | 0.64% | 0.04% | motif file (matrix) |
| 2 | 0.755 | G C T A A G C T C T G A A C T G C T G A C T A G A G C T A C G T C G T A A G T C A G T C | 1e-17 | -40.225729 | 0.70% | 0.07% | motif file (matrix) |
